# Risks of Renal Anomalies and Urinary Tract Infections in Neonates With Single Umbilical Artery

**DOI:** 10.7759/cureus.70876

**Published:** 2024-10-05

**Authors:** Chon In Kuok, Mei Lam Natalie Hsu, Hiu Ching Lam, Wai Hung Chung, Wing Tung Natalie Ho, Choi Kim Judy Kung, Kin Nam Karen Wong, Stephanie Hui Fung Lai, Wei Ling Teresa Ma, Kiu Lok Siu, Winnie Kwai Yu Chan

**Affiliations:** 1 Department of Pediatrics, Queen Elizabeth Hospital, Hong Kong, HKG; 2 Department of Obstetrics and Gynecology, Queen Elizabeth Hospital, Hong Kong, HKG

**Keywords:** congenital anomalies of the kidney and urinary tract (cakut), congenital hydronephrosis, horse shoe kidney, single umbilical artery, urinary tract infection

## Abstract

Background

Our study aims to evaluate the characteristics of congenital anomalies of kidneys and urinary tracts (CAKUT) and urinary tract infection (UTI) in babies with single umbilical artery (SUA) and to identify factors associated with these outcomes.

Methods

We performed a 15-year retrospective analysis on babies who were born ≥34 weeks with SUA between 2006 and 2020. Relevant clinical information on obstetrics and neonatal examinations, UTIs, and imaging of the urinary systems was evaluated.

Results

The frequency of SUA in newborns was 3.28 per 1,000 deliveries. The majority (271/291; 93.1%) of patients had kidney ultrasound, and 19 (7.0%) had CAKUT. Hydronephrosis (n = 11, 57.9%) was the commonest CAKUT, followed by unilateral kidney agenesis (n = 3, 15.8%), horseshoe kidney (n = 2, 10.5%), and right low-lying kidney (n = 2, 10.5%). Most significant CAKUT (including solitary kidney and urinary tract obstruction) could be detected during the antenatal period. Congenital heart defect (OR 4.93, 95% CI 1.59-15.34), limbs (OR 9.77, 95% CI 1.53-62.44), and sacral abnormalities (OR 5.06, 95% CI 1.25-20.55) were associated with CAKUT. Six (2.1%) developed UTIs during the study period, with the majority below two years old. The presence of CAKUT was associated with the development of UTI after adjustment (adjusted HR 10.28, 95% CI 1.86-56.83).

Conclusions

The overall prevalence of CAKUT was 7.0% in patients with SUA, and the majority of significant CAKUT was identified during the antenatal period. Congenital heart defects, limbs, and sacral abnormalities were associated with CAKUT. UTI occurred in 2.1% of patients.

## Introduction

An umbilical cord normally contains two umbilical arteries and one umbilical vein. Single umbilical artery (SUA) is a congenital condition that is characterized by the presence of only one artery in the cord. Previous reviews have demonstrated adverse perinatal outcomes in neonates with SUA, including preterm delivery (OR 2.10), small for gestation age (OR 2.75), and perinatal mortality (OR 2.29) [1,2]. Besides, the presence of SUA was also associated with congenital structural anomalies in different organ systems [3-7].

In particular, the association between SUA and congenital anomalies of kidneys and urinary tracts (CAKUT) has been highlighted in different reviews [3,8]. The reported prevalence of CAKUT in SUA patients ranged from 3% to 11% in various studies [5,6,9-11]. In the past decades, there has been much debate on whether kidney screening ultrasounds are warranted in these newborns. Bourke et al. demonstrated a five-fold increase in CAKUT in SUA patients and advocated screening ultrasound for them [12]. Screening ultrasound and micturating cystourethrography were also recommended to detect vesicoureteral reflux, which appeared to be more prevalent in patients with SUA [13]. On the contrary, some authors questioned the role of screening ultrasound, as many of these congenital anomalies were mild with doubtful clinical significance [8-10]. On top of that, more CAKUT can now be detected with improved antenatal diagnostic accuracy [14]. Further evaluation of the characteristics and progress of these CAKUTs would help decipher the role of postnatal screening ultrasound.

The strong relationship between CAKUT and urinary tract infection (UTI) has been well established [15]. However, given the higher prevalence of CAKUT in SUA, the risk of UTI in this group of patients has not been well delineated in the literature. A comprehensive analysis of factors associated with CAKUT and UTI could fill the current knowledge gap and provide deeper insights into the possible renal involvement in patients with SUA.

In this study, we aimed to determine the prevalence, type, and progress of CAKUT in patients with SUA and to identify the factors associated with its occurrence. UTIs and the associated risk factors in these patients would also be evaluated.

## Materials and methods

Study setting and design

This 15-year retrospective study was conducted in the Department of Pediatrics, Queen Elizabeth Hospital, Hong Kong. Late preterm and term neonates (born ≥34 weeks of gestation) with SUA who were born between January 1, 2006, and December 31, 2020, were included. We excluded the patients if the diagnosis of an SUA was not substantiated in the newborn or placental examinations. The study was approved by the Research Ethics Committee (Kowloon Central/Kowloon East Cluster), Hospital Authority, Hong Kong (approval number KC/KE-21-0123/ER-2).

Data collection

The potential subjects were identified using the International Classification of Diseases, ninth revision (ICD-9) codes 747.5 (absence or hypoplasia of umbilical artery) and 762.6(1) (abnormality of umbilical cord vessel with one artery and one vein affecting fetus or newborn). Relevant clinical information on obstetrics and neonatal examinations, UTIs, and imaging of the urinary systems was extracted from the medical records. Data on or before January 31, 2022, were collected for further analysis.

Newborn assessment

Full physical examinations were performed by pediatric doctors in newborns with SUA. Abnormal physical findings were documented. Since this study focused on congenital abnormalities, physical findings related to birth trauma, such as clavicular fracture and cephalohematoma, were not counted. The classification of abnormal physical findings is summarized in Table 1.

**Table 1:** Categories of congenital anomalies

Category	Examples
Cardiovascular abnormalities	All cardiac structural abnormalities confirmed by echocardiogram
Gastrointestinal abnormalities	Esophageal atresia or tracheoesophageal fistula; anterior displacement of anus or ectopic anus; omphalocele
Limb abnormalities	Digit abnormalities, e.g., hypoplastic digits and polydactyly; skeletal abnormalities, e.g., absent radius
Periauricular abnormalities	Preauricular sinuses; preauricular tags
Sacral abnormalities	Sacral dimples; sacral polyps
Urogenital abnormalities (in males)	Undescended testes; chordee; hypospadias; epispadias

Echocardiograms were performed by pediatric cardiologists based on clinical suspicion, which included the detection of heart murmur and abnormal X-ray findings. Ultrasound of the urinary system and neonatal clinic follow-up were arranged for all neonates with SUA.

Outcome measures

The primary outcome of this study was the type of CAKUT identified in postnatal imaging, while the secondary outcome was the occurrence and characteristics of UTI.

Hydronephrosis was defined as a dilated renal pelvis with a maximum anteroposterior diameter (APD) ≥ 5 mm in renal ultrasonography [16]. UTIs were diagnosed based on (1) positive bacterial culture from a properly collected urine sample, which included (i) >10^5^ colonies forming unit (CFU)/ml of a single uropathogenic in a clean-catch or midstream urine sample or (ii) >10^4^ CFU/ml of a single uropathogenic in a catheterized urine sample, and (2) the presence of clinical features including fever and/or urinary symptoms.

Statistical analysis

Categorical data were expressed as frequencies and percentages and were compared between groups using Pearson chi-square tests or Fisher’s exact tests as appropriate. Continuous variables were expressed as medians and IQRs, and were analyzed by Mann-Whitney U tests. The associations between the factors were explored using binary logistic regression, with estimated ORs and 95% CIs. HRs with 95% CI of risk factors were estimated by Cox proportional hazard models. The time to event was defined as the duration from birth to the development of the first UTI. Significant covariates were further included in multivariate analysis. Statistical analyses were performed using IBM SPSS Statistics for Windows, Version 26.0 (Released 2019; IBM Corp., Armonk, NY, USA). A p-value less than 0.05 was defined as statistical significance.

## Results

Patient characteristics

During the 15-year study period, 28 late-preterm and 263 term neonates (115 boys and 176 girls) with SUA were assessed. The patient characteristics are summarized in Table 2. Most neonates (94.8%) were of Chinese ethnicity. From 2005 to 2020, the overall frequency of SUA in babies ≥34 weeks was 3.28 per 1,000 deliveries (95% CI 2.92-3.68 per 1,000 deliveries).

**Table 2:** Characteristics of patients with SUA (n = 291) GDM, gestational diabetes mellitus; LSCS, lower segment cesarean section; SUA, single umbilical artery; USG, ultrasonography

Parameters	Frequency (%)/median (IQR)
Gender	
Male	115 (39.5%)
Female	176 (60.5%)
Chinese	252 (94.8%)
Singleton	275 (94.5%)
Parity	
1	152 (52.2%)
2	114 (39.2%)
≥3	25 (8.6%)
Gestations (weeks)	38 (37, 39)
Birth weight (grams)	3,010 (2,700, 3,330)
Mode of delivery	
Vaginal delivery	189 (64.9%)
LSCS	102 (35.1%)
Apgar score at one minute	8 (8, 8)
Apgar score at five minutes	9 (8, 9)
Maternal age (years)	32 (28, 36)
Maternal GDM	28 (9.6%)
USG performed	271 (93.1%)

Assessment of CAKUT

Kidney ultrasounds were performed in 271 (93.1%) patients, with the first imaging done at a median (IQR) age of 1.5 (0.5, 2.9) months. CAKUT was detected in 19 (7.0%) patients. Hydronephrosis (n = 11, 57.9%) was the commonest CAKUT, followed by unilateral kidney agenesis (n = 3, 15.8%), horseshoe kidney (n = 2, 10.5%), and right low-lying kidney (n = 2, 10.5%). The degree of hydronephrosis in 10 patients was mild, with a renal pelvic APD measured at 5-6 mm. The remaining patient had severe hydronephrosis with APD 23 mm in the first ultrasound scan. His subsequent MAG3 scan confirmed the diagnosis of pelvic-ureteric junction obstruction (PUJO). Besides, one patient with kidney agenesis also had a duplex kidney on the contralateral side.

All CAKUT patients except one with kidney agenesis received structural scans in the antenatal period. The remaining two unilateral kidney agenesis (n = 2), multi-cystic dysplastic kidney (MCDK) (n = 1), and severe hydronephrosis (n = 1) were detected antenatally. The mild hydronephroses (n = 10), horseshoe kidneys (n = 2), and low-lying kidneys (n = 2) were identified only during the postnatal screening ultrasound.

Factors associated with CAKUT and progress

The patient characteristics between those with and without CAKUT were compared in Table 3. Congenital cardiac abnormalities (OR 4.93, 95% CI 1.59-15.34), limb abnormalities (OR 9.77, 95% CI 1.53-62.44), and sacral abnormalities (OR 5.06, 95% CI 1.25-20.55) were significantly associated with CAKUT.

**Table 3:** Comparison of patient characteristics with CAKUT vs. no CAKUT * For male patients CAKUT, congenital anomaly of kidney and urinary tract; GDM, gestational diabetes mellitus; LSCS, lower segment cesarean section; UTI, urinary tract infection

Parameters	CAKUT (n = 19)	No CAKUT (n = 252)	X^2^ value	p-value
Gender (male)	10 (52.6%)	99 (39.3%)	1.31	0.253
Chinese	18 (94.7%)	241 (95.6%)	0.03	0.59
Singleton	18 (94.7%)	240 (95.2%)	0.01	1
Parity				
1	11 (57.9%)	135 (53.6%)	0.14	0.932
2	7 (36.8%)	101 (40.1%)		
≥3	1 (5.3%)	16 (6.3%)		
Gestations (weeks)	38 (37, 39)	38 (37, 39)	-	0.475
Birth weight (grams)	3,010 (2,710, 3,350)	3,015 (2,710, 3,325)	-	0.861
Mode of delivery				
Vaginal delivery	9 (47.4%)	167 (66.3%)	2.77	0.096
LSCS	10 (52.6%)	85 (33.7%)		
Apgar score at one minute	8 (8, 8)	8 (8, 8)	-	0.489
Apgar score at five minutes	8 (8, 9)	9 (8, 9)	-	0.298
Maternal age (years)	31.5 (26.8, 35.3)	32.0 (29.0, 36.0)	-	0.416
Maternal GDM	0	19 (7.5%)	1.54	0.377
Abnormal newborn exam				
Cardiovascular	5 (26.3%)	17 (6.7%)	9.07	0.012
Gastrointestinal	1 (5.3%)	4 (1.6%)	1.32	0.307
Limb	2 (10.5%)	3 (1.2%)	8.5	0.041
Preauricular	1 (5.3%)	8 (3.2%)	0.24	0.485
Sacral	3 (15.8%)	9 (3.6%)	6.23	0.043
Urogenital*	0	2 (2.0%)	0.21	1
UTI	3 (15.8%)	3 (1.2%)	17.39	0.005

**Figure 1:**
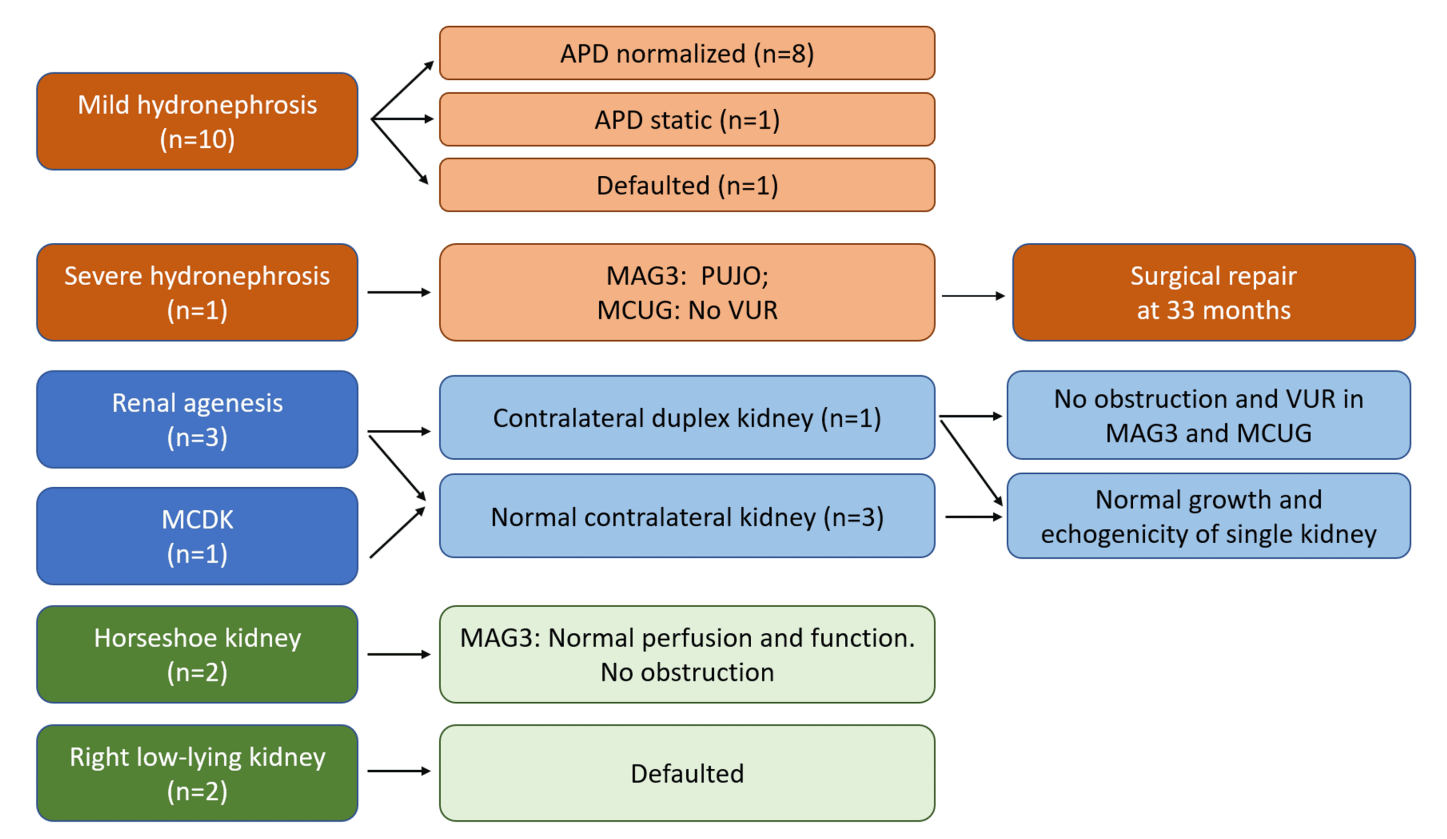
Diagnosis and progress of CAKUT in patients with SUA APD, anteroposterior diameter; MAG3, mercaptoacetyltriglycine radioisotope scan; MCDK, multi-cystic dysplastic kidney; MCUG, micturating cystourethrogram; SUA, single umbilical artery; VUR, vesicoureteral reflux

Follow-up investigations were arranged for patients with CAKUT (Figure 1). Eight out of 10 patients with mild hydronephroses had normalized renal pelvis measurements in the subsequent scans, one had static APD of 5 mm, and one defaulted the follow-up imaging. The patient with severe hydronephrosis and PUJO received laparoscopic pyeloplasty at 33 months. Serial ultrasounds in patients with single-functioning kidneys (n = 4) had normal kidney growth and echogenicity. The MAG3 scans for horseshoe kidneys (n = 2) showed normal functions with unobstructed drainage.

UTI

Six (2.1%) patients developed UTIs during the study period, and half of them had underlying CAKUT. The median age of developing UTI was 10 months old (range: four months to five years old). Five patients had UTIs before two years old, while a patient had a UTI at five years old who presented with fever and dysuria, with positive nitrite and leukocyte esterase from urine dipstick. The coliform organism was isolated from his midstream urine sample. Escherichia coli was the causative agent in the other five patients.

The presence of CAKUT (HR 13.69, 95% CI 2.76-67.87) and congenital heart defects (HR 5.92, 95% CI 1.08-32.32) were significant predictors for the development of UTI in univariate analysis, and CAKUT remained significant after adjustment (aHR 10.28, 95% CI 1.86-56.83). There were no significant associations for other abnormal physical findings and features.

## Discussion

The SUA is defined as the absence of one of the arteries in the umbilical cord and is one of the most common sonographic findings during pregnancy. The frequency of SUA in this study was 3.28 per 1,000 deliveries [4-7], which was comparable to those reported in other centers. Several theories were postulated to explain the development of SUA in the fetus, which include (i) primary agenesis of one umbilical artery, (ii) secondary atresia or atrophy of a previously normal umbilical artery, and (iii) persistence of the original single allantoic artery of the body stalk [1]. However, the exact mechanism remains elucidated.

The association of SUA with congenital anomalies was described in various studies [4-7]. In particular, CAKUT is one of the commonest associations, with a prevalence of around 3-11% [5,6,9-11]. Likewise, our study demonstrated that 7.0% of infants with SUA had underlying CAKUT, which was more prevalent than that in a normal population (3-6 per 1,000 live births) [17]. Based on the higher prevalence, some authors recommended kidney ultrasound screening for early detection of CAKUT in these patients [12,13,18]. However, more recent studies have shown that, despite an apparently higher prevalence of CAKUT, most of these were only minor abnormalities and may have limited clinical relevance. The study by Doornebal et al. showed that four out of five CAKUT detected in SUA patients were only mild hydronephrosis, and the patients remained asymptomatic during the study period [10]. The remaining patient had unilateral hydronephrosis with partial obstruction, which normalized in the subsequent follow-up at three years old. Furthermore, another study by Desphpande et al. demonstrated a similar prevalence of major renal anomalies between infants with SUA and the control cohort and also concluded that postnatal ultrasound was not routinely warranted [19].

Comparatively, our study revealed a higher proportion of clinically significant CAKUT. These CAKUTs, including single-functioning kidneys and horseshoe kidneys, have important clinical implications, as they can be associated with functional abnormalities such as urinary tract obstructions and vesicoureteral reflux and subsequent development of chronic kidney disease [20]. Surgical intervention was required in one of our patients with significant urinary tract obstruction. Apart from our study, more severe CAKUT, such as high-grade vesicoureteral reflux, multi-cystic kidneys, horseshoe kidneys, and kidney agenesis, has also been reported in other patients [8]. Thus, the risk of severe CAKUT in SUA patients could not be underestimated either.

With the advancement in obstetric technology, SUA and CAKUT can be reliably detected during the antenatal period with promising accuracy [3,14]. We further looked into the details of these CAKUTs to see whether these anomalies could be identified antenatally. Our study found that the most severe CAKUT (kidney agenesis, MCDK, and PUJO with severe hydronephrosis) were identified during the antenatal period. However, one surprising finding was that the two patients with horseshoe kidneys were not detected in structural scans. It was known that prenatal diagnosis of horseshoe kidneys could be difficult due to the technical challenges in defining the lower kidney margins and the possible obscuration of the fused isthmus by surrounding bowel echoes [21]. Some additional parameters, such as renal pelvic angle, have been introduced to enhance the sensitivity of prenatal detection of horseshoe kidney [21]. Therefore, incorporating these measurements in checkups for fetuses with SUA may be considered for early diagnosis of this relatively rare CAKUT. In this study, all patients with mild hydronephrosis (APD 5-6 mm in our patients) were undetected antenatally as well; nonetheless, the vast majority of these hydronephroses resolved spontaneously. The benign natural course of these mild hydronephroses may limit the clinical benefit of postnatal screening in patients with normal antenatal scans.

We further explored the risk factors that may predict CAKUT in patients with SUA and identified that congenital heart defects and limb and sacral abnormalities were associated with CAKUT. The congenital anomaly can be an isolated finding; however, approximately 20-30% of children with birth defects actually had multiple congenital anomalies [22]. Specifically, up to one-third of patients with CAKUT also had other extrarenal structural anomalies, with cardiovascular and musculoskeletal systems more commonly affected [23,24]. Besides that, in a group of children with congenital heart disease, Jiang et al. also demonstrated a higher rate of CAKUT at 7.4% [25]. Albeit an increased recognition of collections of congenital anomalies in some patients, the underlying biology of CAKUT and its associations remain to be explored [17,26]. Nonetheless, our findings showed that careful newborn examination and detection of certain congenital anomalies may be helpful in stratifying the risk of CAKUT in patients with SUA.

Our study further explored the features of UTI in patients with SUA, which were not previously evaluated. The reported incidence of pediatric UTI varied among studies, which could be affected by the targeted study population, method of urine collection, and definition of infection. A pediatric review concluded that the overall incidence of UTI in infants was around 0.7% in girls and 2.7% in uncircumcised boys [27]. Our study demonstrated that 2.1% of patients with SUA developed UTIs during the study period. However, in the absence of local pediatric UTI incidence, it is difficult to ascertain whether the presence of SUA increases the risk of childhood UTI. Laboratory investigations showed that Escherichia coli accounted for the majority of uropathogens in our patients, which was in agreement with the local microbiological profile [28]. Our study also showed a higher risk of UTI in patients with CAKUT. Recurrent UTI and CAKUT could result in significant sequelae, including chronic kidney diseases and hypertension [29]. Further research on kidney functions and blood pressure would help delineate the long-term renal outcomes of patients with SUA.

Our study was limited by several factors. Hydronephroses in this study were diagnosed based on the renal pelvic APD. Newer grading systems with consideration of caliceal dilatation and corticomedullary differentiations may improve the accuracy of diagnosis and prognostication [30]. Besides, some possible contributing factors for UTI, such as bowel dysfunction, were not evaluated in the present study.

Despite the limitations, this study systematically reviewed the features and the progress of CAKUT in patients in SUA, with ultrasound evaluation performed in 93.1% of the patients. We identified that most of the significant CAKUT could be identified during the antenatal scans. In addition, newborn examinations, especially detection of cardiovascular, limb, and sacral abnormalities, may be helpful in identifying patients who have a higher risk of CAKUT. To our knowledge, this is the first study that evaluates the risk factors of CAKUT and the characteristics of UTI in patients with SUA. These findings provide a deeper understanding of the possible associated renal complications in patients with SUA.

## Conclusions

CAKUT was present in 7.0% of patients with SUA. The majority of the significant CAKUT (including kidney agenesis, MCDK, and PUJO with severe hydronephrosis) were identified during the antenatal scan. Congenital heart defects and limb and sacral abnormalities were associated with CAKUT. Overall, 2.1% of patients with SUA developed UTIs, with the majority below two years old. Patients with CAKUT had a higher risk of UTIs. Further study is needed to delineate the long-term renal outcomes in patients with SUA.
